# Epigenetic Inheritance: Impact for Biology and Society—recent progress, current questions and future challenges

**DOI:** 10.1093/eep/dvac021

**Published:** 2022-11-05

**Authors:** Rodrigo G Arzate-Mejía, Isabelle M Mansuy

**Affiliations:** Laboratory of Neuroepigenetics, Brain Research Institute, Faculty of Medicine, University of Zurich and Institute for Neuroscience, Department of Health Science and Technology, ETH Zürich, Winterthurerstrasse 190, Zürich CH-8057, Switzerland; Laboratory of Neuroepigenetics, Brain Research Institute, Faculty of Medicine, University of Zurich and Institute for Neuroscience, Department of Health Science and Technology, ETH Zürich, Winterthurerstrasse 190, Zürich CH-8057, Switzerland

**Keywords:** meeting report, epigenetics, epigenetic inheritance, transgenerational inheritance

## Abstract

Epigenetic inheritance has emerged as a new research discipline that aims to study the mechanisms underlying the transmission of acquired traits across generations. Such transmission is well established in plants and invertebrates but remains not well characterized and understood in mammals. Important questions are how life experiences and environmental factors induce phenotypic changes that are passed to the offspring of exposed individuals, sometimes across several successive generations, what is the contribution of germ cells and what are the consequences for health and disease. These questions were recently discussed at the symposium Epigenetic Inheritance: Impact for Biology and Society organized every 2 years in Zürich, Switzerland. This review provides a summary of the research presented during the symposium and discusses current important questions, perspectives and challenges for the field in the future.

## Introduction

In 1942, Conrad Waddington proposed the term “epigenetics,” in the context of developmental biology, to refer to mechanisms of yet unknown nature that allow individual cells to acquire specific morphological and functional features and generate the cellular diversity that characterizes living organisms. The term “epigenetics” meanwhile evolved into a field of research that “studies the molecular mechanisms that can perpetuate alternative gene states in the context of the same DNA sequence” [1]. While much of the work in the field has focused on studying how cells can establish persistent transcriptional states during development and in response to environmental signals and how epigenetic modifications in the genome originate (also called epimutations), the possibility that epigenetic information can flow from one generation to the next has been neglected. Epigenetic inheritance did not immediately appear as a biological phenomenon that could be real in mammals, in part because of the dominant idea that genetics is the sole substrate of heredity and the misconception that germ cells cannot be modified by external factors and receive signals from somatic cells. This vision has changed in the past two decades due to the development of rodent models and techniques to modify, characterize and quantify the epigenome of germ cells. Today, more evidence for epigenetic inheritance has been gained in mammals, and its widespread existence and importance are now well acknowledged. Its underlying mechanisms are however still not understood, nor its impact on health and disease and on evolution in humans.

**Figure 1:**
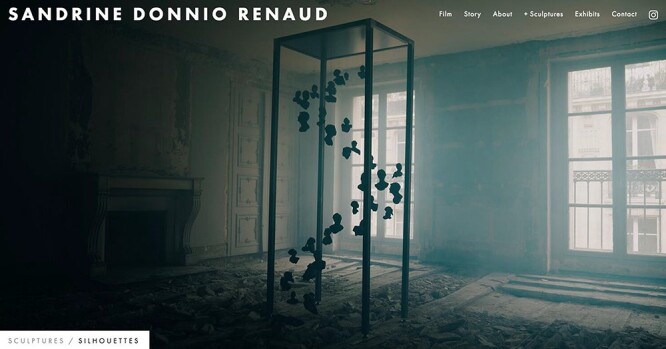
Sculpture “Transgenerational Lineage / DNA Fragment” by artist Sandrine Donnio Renaud, Paris, France https://www.sandrinedonniorenaud.com/. Exhibited at the Brain Research Institute of the University Zürich, Switzerland, Dec 2022.

To approach these complex questions, the field of epigenetic inheritance has capitalized on several disciplines at the crossroad of genetics/epigenetics, reproductive biology, toxicology, neurosciences, medicine, bioinformatics and sociology. To help the field grow and allow people from these different disciplines to meet, exchange and work together, we organize every 2 years since 2017 an international symposium called “Epigenetic Inheritance: Impact for Biology and Society” in Zürich, Switzerland. The third edition of the symposium was held online (due to the coronavirus disease 2019 pandemic) on 25–27 August 2021 and gathered almost 500 participants from all over the world. It had three main sessions covering environmental epigenetics, the power of single-cell technologies and the mechanisms of epigenetic inheritance. It also offered a workshop “Meet the experts” during which current challenges in the field were discussed with a small group of people. As has been the tradition since the first symposium, some art was featured, and this year, we were lucky to have a movie created by the French artist Sandrine Donnio-Renaud called “Transgenerational memory or how the experiences of our ancestors may impact our lives without our knowledge” featuring a sculpture “Transgenerational memory” (Fig. 1) she designed.

This report summarizes the research presented during the meeting (Sessions 1–3), a critical review of the main topics covered and the major points addressed (Conclusions), and potential research lines to advance the field of epigenetic inheritance (Puzzling observations, questions and challenges).

## Session 1: Environmental Epigenetics

The first day of the symposium was devoted to findings on the physiological and molecular effects of environmental exposure in directly exposed individuals and their progeny. The section aimed to illustrate how large epidemiological studies in humans can evidence associations between ancestral exposure and symptomatology in descendants and help provide further support for the potential transmission of acquired traits. Cecilie Svanes (University of Bergen, Norway) presented findings from different large-scale studies evaluating the effects of paternal smoking on the health of descendants. She revisited the concept of windows of susceptibility and highlighted their societal importance to offer intervention opportunities for public health. Prepuberty is a particularly critical but understudied time window during which exposure may induce persistent changes in the maturing germ line with long-term health effects in following generations. Svanes summarized the results of the Respiratory Health in Northern Europe, Spain and Australia cohort, a multigenerational study including 35 000 individuals from 4 generations. In this cohort, the risk of asthma in descendants was significantly higher if the father started smoking before the age of 15 years than if smoking started only before conception. The history of the father’s smoking before the age of 15 years also correlated with lower lung functions in his children and with changes in offspring full blood DNA methylation. The father’s overweight starting prepuberty, independently of overweight in the offspring, increased the likelihood for the offspring to develop nonallergic asthma. Such multigenerational studies highlight how exposure in prepuberty, particularly in the paternal line, may impact health in the offspring with potential consequences for public health.

In the second talk, Adam Wilkins (University of Nottingham, UK) presented data that suggested sperm and seminal fluid differently contributed to embryonic development and may be distinctively implicated in epigenetic inheritance. He showed that a low-protein diet in mice induced a loss of DNA methylation in sperm associated with a downregulation of the expression of DNA methyltransferases (DNMTs) and metabolic genes in testis, suggesting alteration of the testicular environment. Seminal fluid from treated males was also altered and no longer induced the expected inflammatory response of the intrauterine environment during fertilization, indicating a specific effect. However, when both sperm and seminar fluid were used to derive a progeny, all phenotypes are recapitulated, suggesting an additive contribution to embryonic development.

The third talk was presented by Jordana Bell (Kings College London, UK) on the impact of genetic variation on the human methylome. While DNA methylation can vary upon environmental exposure, it is largely influenced by genetic sequences. She found that between 33% and 45% of blood CpG methylome was under local genetic control, e.g. an SNP located within 1 Mb, while the methylation level of 0.7% CpG sites was under effects *in trans*, i.e. SNPs that likely affect genes that influence indirectly the methylation status of CpGs. Remarkably, most of these variable CpGs were located within insulator and enhancer sequences, and their methylation status had high heritability. Interestingly, ∼6% of CpGs were under both local and distal genetic control, and a subset of SNPs had local and distal effects on CpG methylation status, targeting binding sites for transcription factors. Bell pointed out that the influence of genetic variants on the epigenome via differential expression of epigenetic regulators can dominate in some cases, rather than the epigenome being directly changed by external factors. This is however not the primary mechanism in inbred individuals.

The last two talks of the day focused on transgenerational effects of ancestral exposure to toxicants. Emilie Rissman (North Carolina State University, USA) presented an overview of transgenerational effects of bisphenol-A (BPA) on synaptogenesis and behavior. She showed that ancestral exposure to BPA in mice alters social recognition and gene transcription in animals of the third generation (grand-offspring). BPA also increases spine formation at dendrites of neurons in the hippocampus in a dose-dependent manner. Several targets could be identified including estrogen receptors and imprinted genes, e.g. *Meg3*. The transgenerational behavioral and molecular effects were observed in two independent strains of inbred mice (C57BL/6 and FVB), suggesting that BPA affects behavior independently of genetic background.

Then closing the first day, Michael Skinner (Washington State University, USA) presented an overview of 20 years of research in the field of environmental epigenetics with a focus on the toxicant-induced transgenerational susceptibility of disease. He described the inter- and transgenerational effects of endocrine disruptors on physiology and emphasized that the majority of toxicants tested in his laboratory induce pathologies in F1 and F3 generations but have only minor effects in directly exposed individuals (exposure mostly during early embryonic development). As animals from the F3 generation were never directly exposed to the toxicant, such findings suggest a germline-dependent transgenerational origin. Skinner proposed that this likely results from modifications of the epigenome in gametes of exposed individuals that alter gene regulation in the embryo leading to modified transcriptional and epigenetic states in somatic and germ cells of subsequent generations. This ultimately may increase disease susceptibility in following generations. An interesting observation was that transgenerational epigenomic changes induced by toxicants were cell-type specific. Skinner finished his talk calling for an updated view of disease origin and suggested the incorporation of a systems epigenetics perspective. In this context, since environmentally-induced changes to the epigenome of somatic and germ cells can influence gene expression and the emergence of phenotypes, they could also be used as biomarkers of disease predisposition.

## Session 2: The Power of Single-Cell Approaches

The second day of the symposium was more technical and discussed the application of single-cell technologies to gain knowledge on molecular features of mammalian development and characterize the effects of environmental exposure on the epigenome of the germline and offspring. In the first talk, Fuchou Tang (Beijing Advanced Innovation Center for Genomics, China) provided an overview of single-cell methods to explore the regulatory landscape of the human germline. He presented a single-cell sequencing method that allows the simultaneous characterization of the genomic sequence, DNA methylation and the transcriptome of individual cells. Such a multi-omic method is highly innovative and extremely important for the field because it allows the capture of three levels of regulatory information in the same cell at the same time. The genetic sequence (first level) can be used to correlate genetic variants with differences in DNA methylation (second level) that can then be related to the transcriptional activity of genes (third level). Fuchou and colleagues applied this method to assess the safety of maternal spindle transfer in the clinic, a technique to prevent the transmission of mitochondrial DNA mutations from the mother to the offspring. If applied to germ cells during oogenesis and spermatogenesis and to mature gametes, such an approach can help identify and functionally interpret epigenetic changes induced by exposure.

In the second talk, Gavin Kelsey (The Babraham Institute, UK) presented data on the epigenome and transcriptome of oocytes and their modification by environmental exposure. He showed in mice that the oocyte epigenome is hierarchically established, such that transcription instructs the deposition of DNA methylation along gene bodies by recruitment of *de novo* DNMTs via H3K36me3 during elongation. Transcriptional inactivation of specific genes in the oocyte impaired the establishment of DNA methylation at targeted loci, which in turn altered epigenetic and transcriptional regulation in the offspring. This suggests that the epigenome of the oocyte can instruct transcriptional programs in the offspring. Then Kelsey showed the characterization of the epigenome and transcriptome of oocytes from females exposed to high-fat diet (HFD) using single-cell bisulfite sequencing and RNA sequencing (RNA-seq). HFD altered DNA methylation and transcription, although the number of genomic regions with differential methylation was small (<0.2% of all CpG tiles although ∼400 specific differentially-methylated regions could be identified) and changes in messenger RNA (mRNA) abundance were low, suggesting that the oocyte is resilient to chronic HFD. In contrast, oocyte accessory cells (cumulus cells) had a robust transcriptional response to HFD, implying differences in regulatory pathways between oocytes and somatic ovarian cells. HFD also altered 5hmC genome-wide, which may cause transcriptional dysregulation of genes coding for proteins involved in the tricarboxylic acid (TCA) cycle and the production of α-ketoglutare, a major cofactor for demethylases. Since bisulfite sequencing cannot distinguish between 5mC and 5hmC, it could be that the alterations to the epigenome are broader than observed. Kelsey highlighted three intriguing and interesting observations. First, loss of DNMT3A in the oocyte led to aberrant deposition of H3K4me3, suggesting that DNA methylation protects regions from acquiring H3K4me3, which may modify the regulatory landscape of the embryo. Second, exposure to HFD increased transcriptional and epigenetic heterogeneity in oocytes, which otherwise have consistent and structured transcriptome and epigenome in single-cell datasets. The functional effects of such increased variability are unknown, but they might confer some phenotypic plasticity to the next generation when exposed to a salient signal. Third, the transcriptional and DNA methylation profiles of embryos derived by *in vitro* fertilization (IVF) differed from profiles *in vivo*. In particular, IVF induced a developmental delay, an effect that should be considered when comparing embryonic stages of development in IVF- and naturally-derived offspring.

In the following talk, Wolf Reik (The Babraham Institute, UK) further illustrated the power of single-cell approaches to study mammalian development with a technique to analyze spatial transcriptomes (seqFISH) and infer spatial data from single-cell RNA-seq maps. With this method, cell populations with different transcriptional profiles resulting from differential local signaling could be identified. Reik also showed how single-cell multi-omics technologies can help simultaneously interrogate DNA methylation, chromatin accessibility and transcription in individual cells during mouse gastrulation. This multi-level method revealed that pluripotent epiblast cells are epigenetically primed toward an ectodermal fate, with ectoderm enhancers having low DNA methylation and increased chromatin accessibility. Notably, DNA methylation and chromatin accessibility were inversely correlated at regulatory elements during differentiation, suggesting that they influence each other, as previously shown in human cell lines.

Both Kelsey and Wolf discussed how single-cell methods can be used to study the effects of aging on the epigenome in mice and humans. In individual oocytes from old female mice, the method revealed that although methylation was overall lower genome-wide, only a small fraction of CpGs lacked methyl residues, and about a fifth of affected regions was associated with changes in the transcription of neighboring genes. Such differences in DNA methylation and transcription may impact the embryo and explain interindividual differences. In human adult fibroblasts, partial reprogramming by controlled expression of the Yamakana factors resulted in the rejuvenation of the epigenome, the transcriptome and the proteome. DNA methylation at fibroblast-specific enhancers was surprisingly resistant to the partial reprogramming, remained hypomethylated during intermediate states of reprogramming and became fully methylated only in the reprogrammed state. These observations suggest that enhancer epigenetic priming might provide a form of regulatory memory that influences transcriptional programs.

To close the technology session, a short talk by Kai Kleber (Dovetails Genomics, Cambridge, UK) presented different methods offered by Dovetails Genomics to characterize the 3D organization of the genome. For example, Micro-C is a method to detect genome-wide 3D interactions which is based on the digestion of the genome with micrococcal nuclease, an enzyme with sequence-independent endonuclease and exonuclease activity that fragments chromatin more uniformly than restriction enzymes classically used for Hi-C. Other methods of choice are HiChIP and promoter capture conformation which can provide 3D maps of chromatin contacts mediated by specific proteins (HiChIP) or associated with specific genomic regions (promoter capture). By targeting specific sites on chromatin, these methods have better signal-to-noise ratio and higher resolution than Hi-C and allow the characterization of genome-wide regulatory dynamics.

## Session 3: Mechanisms

The last day of the meeting was dedicated to studies of the molecular mechanisms of epigenetic inheritance. In the first talk, Noora Kotaja (University of Turku, Finland) presented data on the effects of paternal HFD exposure on physiology and on the transcriptome of male germ cells in mice. Using a model of HFD in adulthood, she showed that exposed males had increased weight and impaired glucose metabolism but that their progeny (F1), although not affected in basal conditions, gained less weight than the progeny of control males when administered HFD, suggesting a protective response to HFD. Interestingly, the grand-offspring (F2) of HFD fathers had higher insulin levels. Kotaja and collaborators characterized RNA in germ cells, but also in somatic epididymal fat cells of exposed males and their progeny. RNA changes in germ cells were discrete, and for instance, mRNA was not altered in round spermatids of exposed males but only in round spermatids of the offspring after an HFD challenge. However, transfer RNAs-derived small RNAs (tsRNAs) were modified in sperm, consistent with previous reports, and their alterations were partly reversed by normal diet or exercise, suggesting dynamic and not permanent transcriptional changes. Treatment with the antidiabetic metformin after HFD, in contrast, aggravated tsRNA alteration in sperm, raising concern about the possible impact of such a clinical drug in the offspring. In epididymal fat cells, unlike in germ cells, a robust transcriptional response to HFD was observed in exposed males that were fully reversed by a normal diet. RNA was also altered in epididymal fat cells of the progeny but did not correlate with any obvious phenotype.

In the second talk, Anita Öst (Linköping University, Sweden) presented findings that support the involvement of mitochondria, whose role in epigenetic inheritance has hardly been studied in the field until now, in the molecular response of the sperm to a dietary challenge. Using a *Drosophila* model of paternal intergenerational inheritance, her group showed that a varying amount of sugar in the diet altered the transcriptome and proteome of sperm cells. The proteins affected are involved in the regulation of the TCA cycle and in the response to oxidative stress. Increased dietary sugar induced a robust production of reactive oxygen species (ROS) that were demonstrated to be modulated by mitochondria in sperm. In turn, ROS production leads to transcriptional induction of some microRNAs (miRNAs) in sperm, suggesting a link between cellular response to ROS produced by mitochondria and miRNAs. Notably, tsRNAs were also induced by diet in sperm, and most of these tsRNAs were found to be of mitochondrial origin, a feature also observed in human and mouse. An intriguing observation from Öst’s group was that the effects of sugar concentration on ROS induction in sperm were dose-dependent with mid-dosage inducing molecular changes, but low or high dosage having no effect. Such dose-dependency had also been reported with endocrine disruptors and suggests that experiments evaluating the effect of drugs or compounds should be carefully designed and multiple doses need to be tested. Öst finished her talk with preliminary evidence that suggested a role for insulator proteins in the establishment of epigenetic memory in *Drosophila*. Exposure of *Drosophila* embryos to heat shock during the first hour after fertilization resulted in the upregulation of miRNAs and the specific downregulation of *Elba1*, an insulator protein expressed during the first wave of transcription in *Drosophila*. Remarkably, knockdown of *Elba1* compromised the capacity of the cells to establish environmentally-driven changes in the epigenome as evaluated by a position-effect variegation sensor.

In the third talk of the day (short talk), Bermans Iskandar (University of Wisconsin-Madison, USA) discussed the transgenerational effects of folate supplementation in rodents. In contrast to adverse environmental factors, exposure to folic acid in adult rats and mice was beneficial and resulted in increased axonal regeneration capacity of neurons upon injury. Such effect was also observed in the progeny of exposed individuals, and remarkably, it persisted up to the sixth generation of nonexposed animals in an additive manner in each generation. Additive effects across generations were obvious in outbred animals although less marked in inbred strains. The transgenerational effect correlated with changes in DNA methylation and in the expression of genes implicated in axonal growth in the third generation. Interestingly, exposure of both parents to folic acid was necessary to obtain maximal regenerative capacity in the offspring, suggesting that inheritance in this context is equivalent and additively transmitted by both sexes. Finally, Iskandar provided evidence that the regenerative capacity of neurons in nonexposed individuals is a cell-autonomous feature that can be reversed by systemic treatment with histone deacetylase inhibitors or the demethylating agent 5ʹ-azacitidine.

The fourth talk of the session (short talk) by Anara Alshanbayeva, a Ph.D. student in Isabelle Mansuy’s laboratory (University and ETH Zurich, Switzerland), addressed the question of which factors linked to exposure can induce molecular changes in germ cells. The work capitalized on previous findings from the laboratory that serum from stressed males injected intravenously to control males can modify RNA signatures in sperm and cause stress symptoms in the progeny. Alshanbayeva examined if extracellular vesicles (EVs) in blood contributed to these effects and observed that indeed, blood EVs from stressed males injected intravenously alter the sperm transcriptome of injected males. Furthermore, the progeny generated by either IVF or natural breeding had an altered transcriptome at the two-cell stage and metabolic symptoms typical of stressed animals when adult. These results suggest that EVs from the circulation can signal the male germline and be responsible for the transfer of stress phenotypes to the offspring.

Lastly, Brett Nixon (New Castle University, Australia) presented data supporting a role for the epididymis in the induction of molecular changes in sperm. He showed that exposure to acrylamide in mice resulted in changes in the proteome and small noncoding RNA (sncRNA) cargo in epithelial cells of the epididymis and in sperm. The expression of several proteins was increased, e.g. the transcription factor CCCTC-binding factor, and this effect was specific to the caput of the epididymis, suggesting region-specific differences in protein content. The glucocorticoid receptor (GR) was also altered in caput epididymis and correlated with upregulation of miR-20a, miR-30a and miR-152, known to target GR. Besides acrylamide, other types of factors, including heat, altered GR levels and increased its target miRNAs. Acrylamide treatment was detrimental as embryos derived from sperm of exposed males had aberrant transcriptional programs and stopped developing, perhaps partly due to compromised sncRNA cargo.

## Conclusions

### New Concepts

The evolution of a field of research strongly depends on concepts and hypotheses that help a community structure scientific thinking and elaborate experimental plans. The field of epigenetic inheritance was built on the concept that phenotypic traits and features acquired by life experiences or environmental exposure can be transmitted across generations via mechanisms not requiring any change in the genetic sequence. The field grew around the necessity to prove the existence of this form of heredity, particularly in mammals, to identify the underlying mechanisms and study its importance for humans in health and disease. The 2021 edition of the Epigenetic Inheritance Symposium Zürich was extremely useful and constructive in that it brought new results from epidemiological and animal studies, and novel concepts and ideas. One of these concepts is “generational toxicity” proposed by Michael Skinner. Generational toxicity is defined as the property of toxicants to induce or increase the likelihood of disease in descendants of exposed individuals across generations. It derives from the observation that the effects of some toxicants such as endocrine disruptors or of folate can be more pronounced in second- or third-generation individuals than in directly exposed ancestors and that disease phenotypes can emerge only over time. This implies that toxicology studies should not be restricted to exposed individuals and their immediate offspring but need to be extended to further offspring across several generations. The use of machine learning to mathematically analyze the dynamics of epigenetic and transcriptional changes across generations may help extract models of transgenerational transmission and explain complex patterns of epigenetic inheritance. From a public health perspective, the concept of generational toxicity also implies that research initiatives on toxicants, chemicals or drugs should be planned with long-term perspectives to address transgenerational effects.

Another important concept highlighted during the meeting and that emerged in the recent years is the necessity to study multiple levels of epigenetic regulation from DNA methylation and histone posttranslational modifications to transcriptomic changes and chromatin restructuring. Examining only one modification is no longer sufficient, and integrating different layers of epigenetic information has become clearly necessary to be able to identify the factors that can be modified by exposure and the mechanisms of disease etiology and expression. This is however a major challenge because epigenetic information is complex, can be difficult to measure and quantify in germ cells and is variable and can depend on the genetic makeup of each individual. Quantitative multiomics methods with single-cell resolution, refined chromatin techniques such as HiChIP and bioinformatic tools based on machine learning promise to be instrumental for the field in the future. They will allow more precise and efficient profiling, mapping and functional analyses of the epigenome for a better understanding of the mechanisms of epigenetic inheritance.

### New Players in Epigenetic Inheritance

Recent research on the mechanisms of epigenetic inheritance identified several new potential cellular or molecular players that were discussed during the symposium. One of these players is mitochondria in sperm. In *Drosophila*, sperm mitochondria were proposed to be responsible for changes in small RNA composition induced by dietary challenges. Mitochondria are seemingly the only remaining cellular organelles in sperm, present in the flagella and that produce adenosine triphosphate by oxidative phosphorylation to support sperm motility [2]. Mitochondria also generate ROS that are essential for spermatogenesis, sperm capacitation and fertilization, but when in excess, are toxic and impair fertility [3]. Upon dietary challenge, mitochondria alter miRNA expression in sperm through ROS production, but also modify tsRNA populations by differential transcriptional activity. Most tsRNAs reported to be affected by diet in sperm are indeed of mitochondrial origin. Thus, even if sperm mitochondria are eliminated from the embryo at fertilization, they may be affected by environmental exposure and via their metabolic and transcriptional activity that mediate some of the cellular effects contributing to epigenetic inheritance, independently from the nucleus. Since mitochondria are also involved in calcium signal transduction, mitophagy and apoptosis in sperm, their dysfunctions may have multiple consequences and vary depending on the type of exposure. Besides intracellular organelles like mitochondria, extracellular components such as circulating EVs can also contribute to the transmission of phenotypes. This likely involves their cargo (small RNA, proteins and lipids) that, via mechanisms not yet characterized, may transfer signals of exposure to germ cells and likely influence molecular responses of the embryo. It is intriguing to consider that such extracellular microstructures can travel through the body and spread biological signals of exposure to different organs. This concept has however been suggested before [4], but its study in relation to germ cells can provide a new important hypothesis to test.

Other important and notable contributors are somatic cells in gonads and accessory reproductive components. Sertoli cells, epididymal fat cells, nursing cells in the ovary and seminal fluid have been found to be affected by exposure often differently from germ cells, and sometimes more strongly. This suggests that somatic and germ cells have different vulnerability or resilience to external factors, possibly due to distinct membrane receptors and signaling pathways. This may also derive from a different capacity for correction and mechanisms of reversal that may be more prevalent in male germ cells due to their constant self-renewal. This may also depend on the timing of exposure and whether it occurs during prenatal or postnatal development or in adulthood. It is possible that germ cells are less vulnerable in adulthood due to protective mechanisms in gonads such as the blood–testis barrier established by Sertoli cells after birth and the blood–follicle barrier in ovaries. Examining if these barriers are altered by exposure may help understand how germ cells are affected.

### Patterning the Epigenome of Germ Cells

The questions of what the embryo is constituted of at fertilization and how it is shaped during development are major subjects of study in developmental biology. Research in this field is bringing important insight into epigenetic inheritance. For instance, the recognition that gene transcription supports the establishment of DNA methylation in oocytes and affects gene regulation in the embryo implies that transcriptional changes caused by exposure may modify the epigenome of oocytes and the transcriptome of the embryo during critical periods of development. Such regulatory mechanisms could precisely participate in molecular changes underlying generational toxicity and be responsible for the emergence of later disease upon toxicant exposure.

## Puzzling Observations, Questions and Challenges

Finally, we would like to discuss some of the observations and findings reported during the symposium that caught our attention and highlight the questions they raise and the critical areas of study needed to address them.

### About the Susceptibility of the Germline Genome to Epimutations upon Environmental Exposure

*Observation*: Differences in the effects of exposure on the epigenome of germ cells and the possibility that germ cells are differentially susceptible or resilient to exposure.

*Questions to answer*: To what extent are germ cells susceptible or resilient to epigenetic changes induced by exposure? When is the germline epigenome more vulnerable, are there critical windows during development? Does the susceptibility of germ cells depend on the type of exposure, its timing, duration and chronicity? Are female and male germ cells differently affected? Are there mechanisms of resistance, or of correction and reversal and if so, what are they the most effective? Are there differences in susceptibility of the genome in animal models and humans, and what are the implications for evolution?

*Challenges:* Identify the ensemble of changes in the epigenome in female and male germ cells after different exposure and timing. Define possible parameters of resilience. Compare mechanisms across species.

### About the Interplay between Genetic Variation and Epimutations

*Observation*: Genetic variability influences the epigenome, and epimutations can be different depending on the genetic background.

*Questions to answer*: How does interindividual genetic variability affect the establishment of environmentally-induced epimutations?

*Challenge:* Examine genomic and epigenomic profiles simultaneously, when possible, in individual cells. Assess how different genome sequences modulate the establishment and maintenance of epigenetic marks in germ cells, and identify the mechanisms involved.

### About Transcriptional Trajectories of the Offspring of Exposed Individuals

*Observation*: Altered transcriptional profiles in the adult offspring of exposed individuals suggest a deviation from normal regulatory programs that persists throughout life.

*Questions to answer*: Which epigenetic factors in gametes contribute to such “alternative” programs? How do these programs emerge during development? Do they affect most cells or are they established in a cell-type-specific manner? What is the molecular basis for the stabilization and maintenance of such transcriptional states? Are they reversible by environmental interventions?

*Challenges*: Characterize transcriptional profiles across cells in the offspring of exposed individuals. Identify the effect (if any) of epimutations in germ cells on the developing embryo and the regulatory mechanisms involved.

### About the Importance of Biological Components Other Than Germ Cells in Epigenetic Inheritance

*Observation:* Somatic cells such as Sertoli and Leydig cells in testis, epididymal cells, follicles in ovaries as well as noncellular components such as seminal fluid and circulating EVs, which are in direct or indirect contact with germ cells, can be modified (transcriptome, proteome and metabolome) in exposed individuals and/or their progeny.

*Questions to answer:* Can changes in somatic cells or extracellular factors contribute to modifications in germ cells? Do these components depend on the type, timing, duration and chronicity of exposure?

*Challenge:* Dissect the relative contribution of epigenetic information contained in gametes and other cells or noncellular components. Identify differential responses of somatic cells in gonads and determine if they impact germ cells in a way to influence epigenetic inheritance. Assess germ and somatic cells in the progeny. Identify the cellular source of circulating or tissular EVs with an altered cargo due to exposure and clarify if the cargo can be delivered to germ cells directly or indirectly via signaling from intermediate cells.

Since the first symposium in 2017, we witnessed a substantial development in the field of epigenetic inheritance and an increase in the number of research groups working in the field, indicating the importance and interest it gained. While great progress has been made in the past years, evidence still needs to be strengthened and mechanisms better understood in mammals. Major efforts are still needed to move from descriptive to mechanistic analyses and gain causal evidence. Models with more refined and controlled exposure, the use of multiomics approaches and genetic/epigenetic editing based on tools such as Clustered Regularly Interspaced Short Palindromic Repeats (CRISPR) and CRISPR-associated protein 9 (Cas9) are expected to help move the field forward in the near future [5]. We look forward discussing new progress at the next symposium “Epigenetic Inheritance: Impact for Biology and Society” on 23–25 August 2023 in Zürich.

## References

[1] Fitz-James MH , Cavalli G. Molecular mechanisms of transgenerational epigenetic inheritance. *Nat Rev Genet* 2022;23:325–41. 34983971 10.1038/s41576-021-00438-5 PMC7619059

[2] Varuzhanyan G , Chan DC. Mitochondrial dynamics during spermatogenesis. *J Cell Sci* 2020;133:1–11. 10.1242/jcs.235937 PMC7375475 32675215

[3] Park YJ , Pang MG. Mitochondrial functionality in male fertility: from spermatogenesis to fertilization. *Antioxidants* 2021;10:98–121. 33445610 10.3390/antiox10010098 PMC7826524

[4] Berumen Sánchez G , Bunn KE, Pua HH et al. Extracellular vesicles: mediators of intercellular communication in tissue injury and disease. *Cell Commun Signal* 2021;19:1–18. 34656117 10.1186/s12964-021-00787-y PMC8520651

[5] Bohacek J , Mansuy IM. A guide to designing germline-dependent epigenetic inheritance experiments in mammals. *Nat Methods* 2017;14:243–9. 28245210 10.1038/nmeth.4181

